# Complete Genome Sequences of Three of the Earliest Community-Associated Methicillin-Resistant Staphylococcus aureus Strains Isolated in Remote Western Australia

**DOI:** 10.1128/MRA.00797-21

**Published:** 2021-09-16

**Authors:** Nicola M. Karakatsanis, Elena Colombi, Shakeel Mowlaboccus, Julie C. Pearson, Geoffrey W. Coombs, Joshua P. Ramsay

**Affiliations:** a Curtin Health Innovation Research Institute, Curtin University, Bentley, Western Australia, Australia; b Curtin Medical School, Curtin University, Bentley, Western Australia, Australia; c College of Science, Health, Engineering, and Education, Murdoch University, Murdoch, Western Australia, Australia; d Department of Microbiology, PathWest Laboratory Medicine WA, Fiona Stanley Hospital, Murdoch, Western Australia, Australia; University of Rochester School of Medicine and Dentistry

## Abstract

Initially reported in Western Australia in the 1980s, community-associated methicillin-resistant Staphylococcus aureus (CA-MRSA) has become a major cause of S. aureus infections globally. We report the complete genome sequences of three of the earliest CA-MRSA strains isolated from remote Australian Indigenous communities in the Kimberley region of Western Australia.

## ANNOUNCEMENT

Methicillin-resistant Staphylococcus aureus (MRSA) is a leading cause of health care-associated infections worldwide and has emerged as a major cause of infections in the community (1). Community-associated MRSA (CA-MRSA) is genetically distinct and was first detected in Western Australia in 1984, in remote Indigenous communities (2, 3). Here, we present the complete genome sequences of three of the earliest isolated CA-MRSA strains, i.e., WBG7583 (sequence type 8 [ST8]), WBG8381 (ST5), and WBG8366 (ST78) (4, 5).

Previously isolated S. aureus strains (4) were rehydrated from lyophilized stocks in tryptic soy broth (TSB) and grown overnight on blood agar at 37°C. Strains were grown overnight in 5 ml TSB at 37°C, and cells were harvested by centrifugation. Cells were resuspended in 200 μl Tris-EDTA (TE) buffer containing 1 mg/ml RNase (Sigma-Aldrich, USA) and 100 μg/ml lysostaphin (Sigma-Aldrich) and were incubated at 37°C for 10 min. DNA for Oxford Nanopore Technologies (ONT) MinION sequencing was extracted using the FavorPrep blood/cultured cell genomic DNA extraction minikit (product number FABGK-001-2; Favorgen Biotech Corp., Taiwan). For Illumina NextSeq sequencing, DNA from a separate culture was extracted using the DNeasy blood and tissue kit (product number 69506; Qiagen, Germany). No DNA shearing or size selection was carried out before sequencing.

Genomes were sequenced using the MinION Mk1B system (ONT, UK). The ONT SQK-RAD004 kit was used to create DNA sequencing libraries, which were loaded onto an ONT FLO-MIN106 flow cell and sequenced using ONT MinKNOW v20.10.3 and MinKNOW Core v4.1.2. Base calling was performed with Guppy v4.4.1+1c81d62 (ONT), using the dna_r9.4.1_450bps_hac.cfg model. NanoFilt (6) was used to filter reads for size (>4 kb) and average quality (scores of >Q10), and genomes were assembled using Flye v2.8.3-b1695 (7). Unfiltered MinION reads were mapped to each genome assembly with Minimap2 v2.17-r941 (8) and used to polish the assembly six times with Racon v1.4.15 (github.com/lbcb-sci/racon). Each genome was also sequenced with the Illumina NextSeq 500 platform using the Nextera XT DNA library preparation kit (150-bp paired-end chemistry) and the NextSeq 500/550 kit v2.5 (300-cycle format; Illumina, USA). Nesoni clip (github.com/Victorian-Bioinformatics-Consortium/nesoni) was used to remove adaptor sequences and to quality filter the reads. Illumina reads were mapped to the genome assembly with Minimap2 v2.17-r941 (8), and mapped reads were used to polish the assembly five times using Pilon v1.23 (9). Circlator v1.5.5 (10) was used to set the start position of each genome, and Qualimap v2.2.2-dev (11) was used to generate genome statistics (Table 1). Genome annotation was performed using the NCBI Prokaryotic Genome Annotation Pipeline (PGAP) v5.1 (12). Default parameters were used for all software unless otherwise specified.

**TABLE 1:** Genome and mapped-read statistics for WBG7583, WBG8381, and WBG8366

Strain and replicon type	Genome size (bp)	GC content (%)	GenBank accession no.	Data for MinION sequencing				Data for Illumina sequencing			
				Total no. of reads (mapped)	Read *N*_50_ (bases)	Mean read depth (×)	SRA accession no.	Total no. of reads (mapped)	Mean read length (bp)	Mean read depth (×)	SRA accession no.
WBG7583 (ST8)				1,139,489	2,373		SRX11246052	3,279,211	147		SRX11246051
Chromosome	2,790,388	33	CP070989.1			593				100	
pWBG753	30,029	30	CP070990.1			2,061				232	
WBG8381 (ST5)				1,900,172	2,493		SRX11247744	2,400,046	147		SRX11247745
Chromosome	2,820,507	33	CP071046.1			1,005				62	
Circular prophage WBG8381	42,493	35	CP071049.1			2,226				151	
pWBG749	38,087	30	CP071047.1			2,312				193	
pWBG8381	2,539	31	CP071048.1			31,299				409	
WBG8366 (ST78)				189,095	3,057		SRX11246056	1,880,412	148		SRX11246055
Chromosome	2,768,386	33	CP070983.1			114				62	
pWBG763	20,730	28	CP070984.1			516				213	
pWBG764	2,393	28	CP070985.1			11,503				330	

### Data availability.

GenBank accession numbers for the genome assemblies are provided in Table 1. The MinION and Illumina sequencing reads have been deposited under BioProject accession numbers PRJNA703734 and PRJNA703736 and Sequence Read Archive (SRA) accession numbers SRX11246052, SRX11246051, SRX11247745, SRX11247744, SRX11246056, and SRX11246055, as indicated in Table 1.
